# COVID-19 cases with delayed absorption of lung lesion

**DOI:** 10.1515/med-2021-0265

**Published:** 2021-04-27

**Authors:** BinBin Li, WeiZhen Hu, ChunMiao Bao

**Affiliations:** Department of Infection, YongJia County People’s Hospital, Shangtang Road, Wenzhou, China; The Affiliated Yangming Hospital of Ningbo University, Yuyao People’s Hospital of Zhejiang Province, Yuyao, China

**Keywords:** cox regression, survival analysis, COVID-19, computed tomography, SARS-CoV-2

## Abstract

**Objective:**

Over 90% of the COVID-19 patients with computed tomographic (CT) manifestations showed radiological improvement on dissipating stage. Cases with refractory pulmonary infiltration were discussed in this study.

**Methods:**

During hospitalization, chest CT scan and reverse transcriptase polymerase chain reaction (RT-PCR) test were repeatedly performed. While drawing parallels to RT-PCR, the impact of delayed absorption of lung lesions on length of hospital stay (LOS) and medical expense was investigated. Features for delayed absorption of lung lesions were identified using cox proportional hazard regression model.

**Results:**

Cases with delayed absorption of lung lesions had a prolonged LOS (18.00 ± 4.90 vs 9.25 ± 4.20, *p* < 0.01) and increased medical expense (9124.55 ± 2421.31 vs 4923.88 ± 2218.56, *p* < 0.01). Time interval from admission to a negative RT-PCR (ATN) was also prolonged (13.29 ± 4.72 vs 9.25 ± 4.20, *p* = 0.03). The cox proportional hazard regression model indicated that imported cases bore high risk of delayed absorption of lung lesions (hazard ratio = 2.54, 95% confidence interval 1.05, 6.11, *p* = 0.04). Sensitivity analysis revealed similar pattern (hazard ratio = 6.64, 95% confidence interval 1.62, 27.18, *p* = 0.01).

**Conclusion:**

Imported cases of COVID-19 were more likely to have refractory pulmonary infiltration, which subsequently prolongs LOS and increases medical expense.

Chest computed tomography (CT) plays an important role in the early detection of COVID-19 pneumonia, monitoring clinical progression, evaluation of disease severity, and examining the recovery [1]. Patients with COVID-19 had high viral load near presentation, which produced a positive result of reverse transcriptase polymerase chain reaction (RT-PCR) at the early phase but varied at the intermediate stage and turned into negative RT-PCR at the late phase of infection [2]. The CT image and RT-PCR do not always appear parallel on COVID-19 patients. Tao Ai and his colleagues reported that in the 413 patients with negative test result of RT-PCR, 308 of 413 (75%) had lung infiltration in CT scan [3]. This study was aimed to evaluate the impact of delayed absorption of lung lesions on medical resource burden and identify the possible feature for this refractory pulmonary infiltrate.

## 1 Methods

Study population was collected from a village. Individuals with confirmed diagnosis of COVID-19 with RT-PCR were consecutively recruited. Demography (age, gender, and body mass index [BMI]), transmission routes (imported from epicentre vs locally transmitted), and other variables (comorbidity, smoking status, and time interval from symptom onset to admission [STA]) were collected during epidemiological investigation. Diabetes and hypertension were reported as comorbidities, whereas no other comorbidity was found in this study population. Time interval from STA was used to measure the timing of medical intervention. Longer STA indicates a delayed medical intervention. During the hospitalization, chest CT scan and RT-PCR test were performed every 3 days. Bilateral pulmonary infiltration was found in each COVID-19 case in this study. A triple combination of IFN α-2b, lopinavir, and umifenovir was initiated in each patient after admission, while no therapy was given before the hospitalization. Laboratory tests, time interval from admission to negative RT-PCR (ATN), length of hospital stay (LOS), and medical expense were collected. ATN was used to measure the change of viral load. Longer ATN indicated a prolonged coronavirus shedding. Patients with a negative test result of RT-PCR while CT scan showing lung lesion were defined as cases with delayed absorption. This study was approved by the research ethics committee of Yongjia County People’s Hospital (approval number: 2020-L01), with a waiver of informed consent.

Intergroup differences (delayed absorption of lung lesions vs control) were assessed using independent sample *t* test, Wilcoxon signed rank tests, or chi-square test. Age, gender, BMI, transmission routes (imported vs local), comorbidity, smoking status, and STA were included in the cox proportional hazard regression model to identify the possible feature for delayed absorption of lung lesion. Sensitivity analysis was performed by including laboratory test in the cox regression model. All analyses were performed with STATA 16.0 (Stata Corp, College Station, TX, USA), and *p*-value < 0.05 was defined as statistically significant.

## 2 Results

All patients (*n* = 46) in this study were fully recovered from COVID-19 without ventilation support. Epidemiological investigation identified 14 imported cases travelled back from epicentre and 32 locally transmitted cases. Patients with delayed absorption of lung lesions had a significantly prolonged LOS (18.00 ± 4.90 days vs 9.25 ± 4.20 days, *p* < 0.01; Table 1) and higher medical expense (9124.55 ± 2421.31 CNY vs 4923.88 ± 2218.56 CNY, *p* < 0.01; Table 1). In addition, ATN was also delayed (13.29 ± 4.72 days vs 9.25 ± 4.20 days, *p* = 0.03; Table 1). After adjusted for age, gender, BMI, comorbidity, and smoking status, cox regression model identified the role of transmission route as a prognostic factor, suggesting that imported cases bore high risk of refractory lung damage (hazard ratio = 2.54, 95% confidence interval 1.05, 6.11, *p* = 0.04; Table 2). Sensitivity analysis showed similar result (hazard ratio = 6.64, 95% confidence interval 1.62, 27.18, *p* = 0.01; Table 2), indicating the robustness of our finding.

**Table 1:** Comparison between cases with delayed absorption of lung lesions and non-delayed cases

	Total	Delayed absorption of lung lesions	Control	*p*-value
	(*N* = 46)	(*N* = 38)	(*N* = 8)	
Age^a^	43.85 (14.32)	45.47 (14.24)	36.13 (12.82)	0.09
Male^b^	23 (50.0)	19 (50.0)	4 (50.0)	1.00
BMI^a^	22.12 (1.88)	22.01 (1.78)	22.54 (2.33)	0.49
Imported^b^	14 (30.4)	12 (31.6)	2 (25.0)	0.71
Comorbidity^b^	9 (19.6)	7 (18.4)	2 (25.0)	0.65
Smoke^b^	5 (10.9)	4 (10.5)	1 (12.5)	1.00
STA (day)^c^	3.0 (4.0)	3.0 (4.0)	2.0 (1.0)	0.32
ATN (day)^a^	12.59 (4.84)	13.29 (4.72)	9.25 (4.20)	0.03
LOS (day)^a^	16.48 (5.81)	18.00 (4.90)	9.25 (4.20)	<0.01
Medical expense (CNY)^a^	8262.87 (2913.42)	9124.55 (2421.31)	4923.88 (2218.56)	<0.01

Abbreviations: STA – symptom to admission; ATN – admission to negative reverse transcription polymerase chain reaction; LOS – length of hospital stay; CNY – Chinese Yuan.

a Mean (SD).

b *n* (%).

c Median (IQR).

**Table 2:** Multivariate cox regression model

	Without laboratory test			With laboratory test		
	HR	*p*-value	95% CI	HR	*p*-value	95% CI
Imported	2.54	0.04	1.05, 6.11	6.64	0.01	1.62, 27.18
Age	0.96	0.06	0.92, 1.00	0.93	0.02	0.88, 0.99
Male	1.37	0.47	0.58, 3.24	1.08	0.93	0.22, 5.31
BMI	0.93	0.56	0.73, 1.19	0.93	0.68	0.65, 1.33
Smoke	1.15	0.83	0.34, 3.87	1.88	0.40	0.44, 8.11
Comorbidity	0.63	0.50	0.16, 2.42	1.96	0.47	0.31, 12.27
STA	1.09	0.24	0.94, 1.27	1.08	0.38	0.91, 1.27
WBC				1.15	0.39	0.84, 1.57
Plt				1.00	0.59	0.99, 1.01
CRP				0.97	0.07	0.94, 1.00

Abbreviations: STA – symptom to admission; HR – hazard ratio; CI – confidence interval; WBC – white blood cell; Plt – platelet; CRP – c-reactive protein.

## 3 Discussion

CT scan was extensively applied in early diagnosis of COVID-19 [4]. As compared to viral load shedding, patients with delayed recovery in lung damage were studied to identify the possible feature to deteriorate prognosis. In this study, patients with delayed absorption of lung lesions may substantially prolong LOS and raise medical expense, which increases medical resource use burden on the healthcare system. The difference in demography and medical history between delayed absorption and control was not statistically significant, suggesting that age, gender, and comorbidity were not likely contributing to the prolonged hospital stay and extra medical expense. Under the national policy, all medical expenses in treating COVID-19 were covered by healthcare insurance in China, in which case, it is not likely that medical expense would be affected by social-economic status. As a measurement for conversion of RT-PCR, ATN was found prolonged on patients with delayed absorption of lung lesions, suggesting that the difference in chest CT scan is not only a reflection of later phase of lung tissue repair but also an indication of delayed viral load shedding.

Previous studies have shown that demographic factors [5,6], comorbidity [7], and smoking status [8] were associated with the development of COVID-19. Therefore, covariates of age, gender, BMI, comorbidity, and smoking status were included in our model. After adjusting these potential confounders, high risk of deteriorating prognosis in imported cases was identified in this study, which supports a stratified processing of COVID-19 patients based on transmission routes. Similar result was reported in several published studies. By comparing the dynamics of viral load between imported and local COVID-19 patients, a gradual decrease in the infectivity of SARS-CoV2 in tertiary patients was found [9]. Clinical descriptions of COVID-19 showed a higher proportion of severe cases (requiring invasive mechanical ventilation) in the early stages of pandemic outbreak [10]. Possible reasons causing the high risk of delayed absorption of lung lesions in the imported cases may relate to subtypes of virus. An investigation of molecular divergence between SARS-CoV-2 subtypes identified two major types: L type and S type [11]. L type is a more virulent subtype with high frequency in the epicentre but lower frequency in other areas, whereas the S type is less aggressive but has increasing frequency in non-epicentre areas. In addition to previous studies, we presented radiographic material of refractory pulmonary infiltration, warranting a more complex follow-up strategy for imported cases of COVID-19.

The strength of this study is multifold. This study identified an association between refractory lung damage and high burden of medical resource, which suggests that a sensitive modality like CT might be beneficial to speed up therapeutic workflow. On the basis of the epidemiological investigation, travel trace of patients could be used to improve risk stratification, without waiting for the results of the swab test. This study also has several limitations. Transmission route was set as a binary variable (imported vs locally transmitted). Furthermore explicit stratification (secondary, tertiary generation) was not feasible in this study. Another limitation was the variable sensitivity of RT-PCR, which relied on the proper collection of nasopharyngeal swabs and sputum samples.
